# Fatal retroperitoneal hematoma associated with Covid-19 prophylactic anticoagulation protocol

**DOI:** 10.1016/j.radcr.2021.04.029

**Published:** 2021-04-15

**Authors:** Michael Teta, Michael J Drabkin

**Affiliations:** a Critical Care, Catholic Health, Long Island, New York; b Department of Radiology, Catholic Health, Long Island, New York

**Keywords:** Retroperitoneal Hematoma, Anticoagulant therapy, Lovenox, Covid-19, Embolization

## Abstract

Due to the association between Covid-19 and thromboembolic events, there has been a surge in anticoagulation use during the pandemic based on evolving guidelines for management of hospitalized Covid-19 patients. Spontaneous soft tissue hematoma can be a severe complication of anticoagulation. Herein we present a fatal case of severe spontaneous soft tissue hematoma secondary to anticoagulant therapy in a 67kg 81-year-old female with chronic kidney disease who was admitted to the hospital with Covid-19 pneumonia. There is currently no evidence of mortality benefit among Covid-19 patients on high-dose anticoagulation. In the future we hope that practitioners will consider the bleeding risks of anticoagulation and consider patients’ age, weight and renal function when determining prophylactic anticoagulation regimens in Covid-19 patients.

## Introduction

Spontaneous soft tissue hematoma (SSTH) can be a severe complication of anticoagulation use [1,2]. Incidence of SSTH is rising with increasing use of anticoagulation [2,3]. SSTHs occur most frequently in the rectus sheath and iliopsoas but can occur in other locations as well [2]. Most cases are self-limited and resolve with cessation of anticoagulation and conservative management [4]. The high-rate of success of conservative management is largely due to the fact that these hematomas occur in contained spaces which allows them to be tamponaded by adjacent structures [4]. In certain cases, particularly when coagulopathy cannot be easily reversed or if there is a delay in diagnosis, the SSTH can grow to the point that it causes tearing of additional vessels. In such cases there is a high rate of mortality [1]. In these cases of persistent bleeding or hemodynamic instability despite conservative management, transcatheter arterial embolization (TAE) is generally the next line of treatment; in rare cases surgical management is pursued [1,2].

During the recent pandemic, there have been large numbers of Covid-19 patients affected by thromboembolic events, occurring in 11-70% of ICU patients with Covid-19 [[5], [6], [7], [8]]. In response to this, new and more aggressive anticoagulation guidelines have emerged for management of hospitalized Covid-19 patients [9]. As of the writing of this paper, greater than 100 million people have been diagnosed with Covid-19 [10]. Given the scale of Covid-19, any related treatment guidelines can impact large numbers of patients. Many of the existing prophylactic anticoagulation guidelines recommend 40mg of Lovenox once daily for Covid-19 patients with mild symptoms and 0.5 mg/kg of Lovenox twice daily for patients with more severe symptoms or patients that require ICU admission [9,11]. Initiation of therapeutic anticoagulation of 1mg/kg of Lovenox twice daily has been suggested for high-risk patients who require ventilator support or have a markedly elevated D-dimer [9,11].

As we prescribe these anticoagulants for our patients, we must understand the associated risks. In the general population 0.9-16.5% of patients experienced a major bleeding episode while on anticoagulation therapy [6,[12], [13], [14]]. Higher doses of Lovenox have been associate with increased rates of bleeding complications in the general population as compared to lower doses [15].

A recent study evaluating anticoagulant use in 42 Covid-19 patients found no clinical benefit to the use of therapeutic anticoagulation in Covid-19 patients, however, the same study also did not find any increased incidence of bleeding in these patients [16]. One recent case report described a major bleeding complication from full dose anticoagulation in a Covid-19 patient which resulted in significant morbidity but not mortality [17]. We present a case which is to the best of our knowledge the first reported mortality occurring secondary to bleeding resulting from anticoagulation use in a Covid-19 patient.

## Case presentation

An 81-year-old female weighing 67kg with a past medical history of hypertension, hyperlipidemia, hypothyroidism, COPD and 40 pack-years of smoking, presented to the emergency room with shortness of breath. She was found to have Covid-19 pneumonia and admitted for management of hypoxic respiratory failure. Biomarkers upon admission included a ferritin of 1,462 ng/mL (reference range 12-150), creatine kinase of 114 U/L (reference range 30-135), procalcitonin of 0.56 ug/L (reference range <0.5), D-dimer of 21 mcg/mL (reference range <0.4), and C-reactive protein of 63 mg/L (reference range 0.3-10). Her platelet count (reference range 150-400 × 10(3)/mcL) and hemoglobin (Hgb) (reference range 11.5-17.3 g/dL) were within normal limits and her creatinine clearance was 29 mL/min (reference range >60). As per hospital Covid-19 protocol, she was started on an elevated prophylactic dose of 40mg of Lovenox twice daily (BID). She took Aspirin 81 mg daily at home and was started on Aspirin 325 mg in the hospital. Her respiratory status was initially stable on nasal cannula oxygen.

On hospital day 5 she experienced hypoxia which resulted in her transfer to the medical intensive care unit. The patient's Lovenox dose was empirically increased to 60 mg BID out of concern for pulmonary embolism (PE). Subsequent CT angiography (CTA) of the chest was negative for PE, with incidental findings of a small amount of fluid in the LUQ of unclear etiology (seen only on the very last slice of the CTA). Later that day, the patient became obtunded, pale, and hypotensive. She was intubated for airway protection, and developed shock requiring vasopressors. Arterial blood gas demonstrated profound acidosis and Hgb of 3.7 g/dL. This was a large drop from Hgb of 11.5 g/dL just 20 hours prior. Her lactic acid was 19.0 mmol/L (reference range 0.4-2.0). Physical exam, including nasogastric lavage, showed no obvious source of bleeding. Her abdomen was soft, nontender, nondistended. After transfusion of 2 units of packed red blood cells (pRBCs), her Hgb improved to 8.5 g/dL. However, the patient continued to require pressor support and transfusion of additional blood products. All anticoagulants and antiplatelet agents were stopped.

**Fig. 1:**
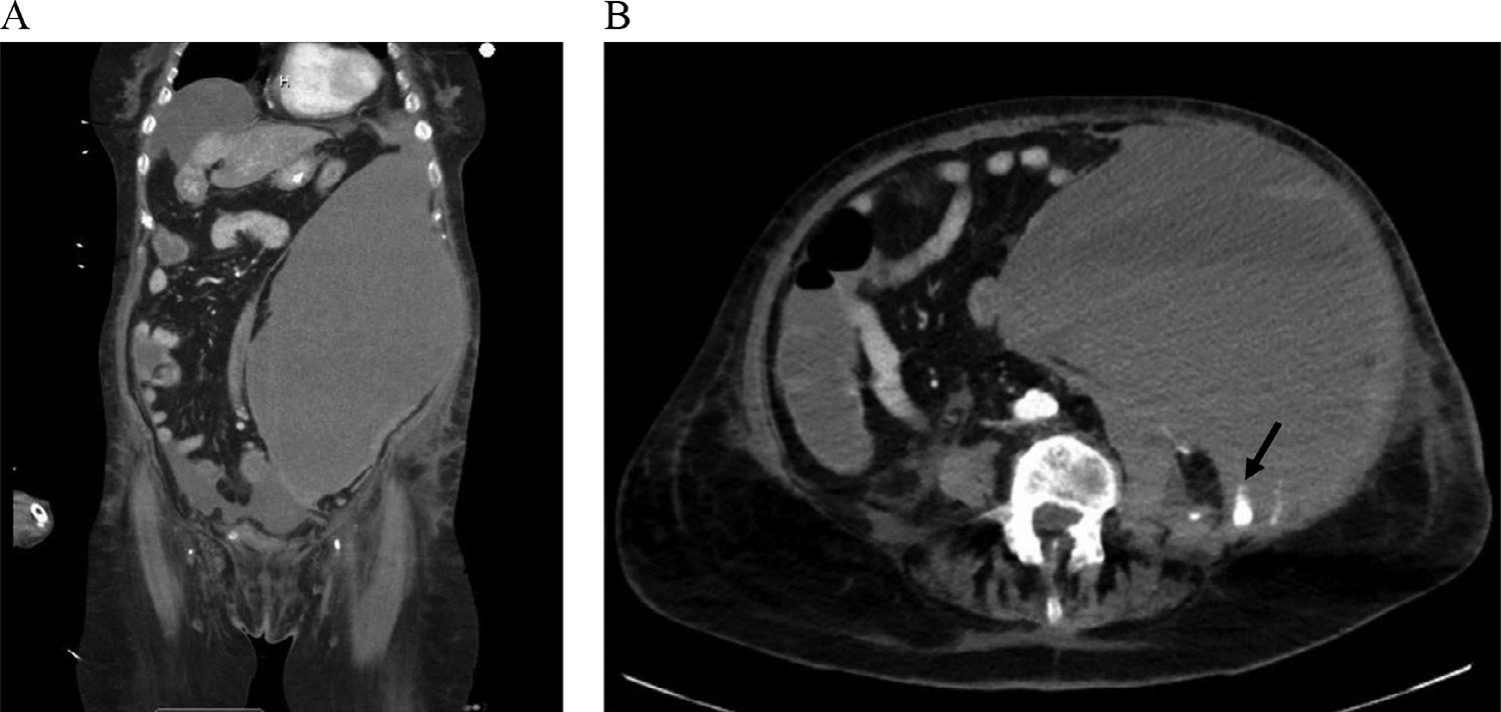
(A) Coronal CT Angiography image through abdomen demonstrates a very large retroperitoneal hematoma. (B) Axial CT Angiography image through the mid abdomen demonstrates a large blush of active extravasation of contrast as well as several smaller blushes within the hematoma.

**Fig. 2:**
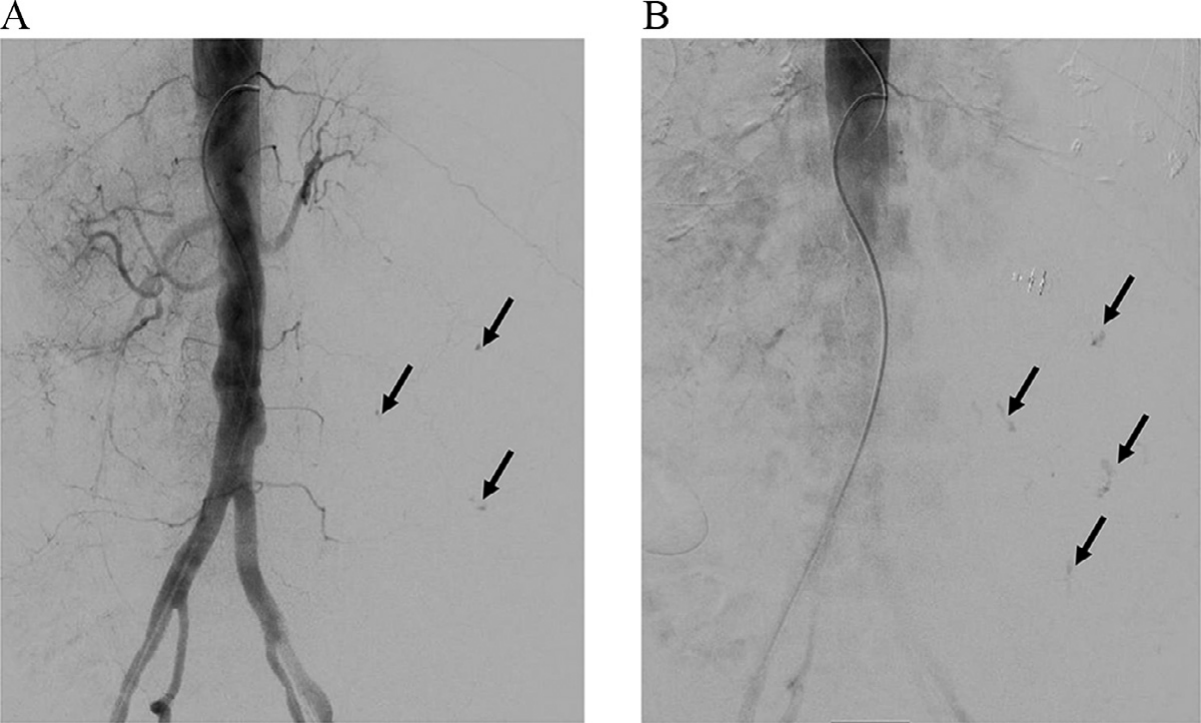
(A) Aortography demonstrates contrast extravasation from several lumbar arteries. (B) Delayed imaging demonstrates increase and persistence of multiple areas of active extravasation.

**Fig. 3:**
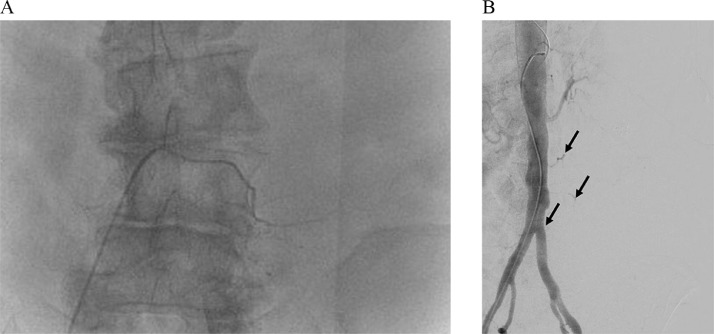
(A) Selective angiography and embolization of the lumbar arteries. (B) Completion aortography shows stasis of contrast within the 3 embolized lumbar arteries. The active extravasation seen on initial aortography is no longer present.

On day 6 she further destabilized and required greater volume of blood products. The patient developed multi-organ failure secondary to profound hypoperfusion evidenced by derangements on complete metabolic panel and worsening coagulopathy. Non-contrast CT performed late that night demonstrated a large left retroperitoneal hematoma measuring up to 25cm in greatest diameter. CTA performed in the early morning hours of hospital day 7 demonstrated active extravasation of contrast into the expanding left retroperitoneal hematoma, now measuring up to 31cm (Fig. 1). That morning she was then taken to interventional radiology and had technically successful embolization of multiple bleeding lumbar arteries (Figs. 2 and 3). She was briefly stabilized during the day, but that night experienced another large drop in Hgb from 11.5 g/dL to 5.0 g/dL and died in the early hours of hospital day 8.

## Discussion

Due to the association between Covid-19 and thromboembolic event, there has been a surge in anticoagulation use during the pandemic based on evolving guidelines for management of hospitalized Covid-19 patients. In the present case we have a severe SSTH which developed secondary to prophylactic and/or empiric anticoagulation in a Covid-19 patient. Despite supportive care and TAE, this hemorrhage ultimately resulted in death.

As anticoagulant therapy guidelines for Covid-19 patients are evolving, it is important to remember that anticoagulant use is not without risk. Hemorrhagic complications of anticoagulant therapy are relatively common in hospitalized patients, and although most of these are self-limited, morbidity and mortality does occur [[1], [2], [3], [4]]. Furthermore, it is important to keep in mind that Lovenox is renally cleared. The patient in our case had a creatinine clearance of only 29 mL/min; in patients with impaired renal function dose adjustment for Lovenox should be considered and Anti-Xa monitoring should be performed [18]. Aspirin use should also be taken into account as combined use of antiplatelet and anticoagulants can increase bleeding risk.[19]

Initially part of the rationale for empiric high-dose anticoagulation therapy in Covid-19 patients was to avoid CT imaging entirely due to patient instability and concern regarding maintaining isolation precautions. A potential solution would be to screen Covid-19 patients for DVT with lower extremity ultrasound. This can help lead to earlier detection of deep venous thrombosis and more precise use of anticoagulants [20].

At the present time there is no significant evidence of mortality benefit among Covid-19 patients on high-dose anticoagulation. As we write this case report, anticoagulation trials for Covid-19 patients (REMAP-CAP, ACTIV-4, and ATTACC) have been halted due to futility and safety concerns [21].

We hope that this case report will serve as a reminder of the inherent risks of anticoagulant therapy and may steer clinicians away from high-dose prophylactic doses or initiation of empiric therapeutic anticoagulation. When a SSTH does occur, anticoagulation should immediately be halted and if necessary reversed. In severe cases interventional radiology should be consulted for TAE.

This case highlights the continued need to practice evidence-based medicine. Despite the terrible and unusual situation that the Covid-19 pandemic has created, we should adhere as closely as is practical to our guiding principles. There is currently no evidence of mortality benefit among Covid-19 patients on high-dose anticoagulation and literature regarding anticoagulation in the general population has shown that there is increased bleeding risk. Further research is needed to assess the efficacy and safety of various doses of anticoagulant therapy in Covid-19 patients.

## Declaration of interests

The authors declare that they have no known competing financial interests or personal relationships that could have appeared to influence the work reported in this paper.

The authors declare the following financial interests/personal relationships which may be considered as potential competing interests:

The authors have no conflicts of interest.

The authors have not received any outside funding.

This work has not been previously published or presented in any format.
